# 4172 Introduction to R Programming and GitHub: Developing Automated Analysis of Complete Blood Count Data as a Translational Science Undergraduate Project

**DOI:** 10.1017/cts.2020.215

**Published:** 2020-07-29

**Authors:** Jeffrey Robinson, Annica Wayman

**Affiliations:** 1 National Institutes of Health; 2 UMBC Translational Life Science Technology Bachelors Program

## Abstract

OBJECTIVES/GOALS: Introduce students to programming and software development practices in the life sciences by analyzing standard clinical diagnostic bloodwork for differential immune responses. Including lectures and a semester project with the goal of enhancing undergraduate students’ education to prepare them for careers in translational science. METHODS/STUDY POPULATION: The educational content was taught for the first time as a component of the newly developed course BTEC 330 “Software Applications in the Life Sciences” in UMBC’s Translational Life Science Technology (TLST) Bachelor’s degree program at the Universities at Shady Grove campus. Eleven students took the course. All were beginners with no programming background. Lectures provided background on the diagnostic components of the CBC, criteria for differential diagnosis in the clinical setting, and introduction to hematology and flow cytometry, forming underpinnings for interpretation of the CBC results. Weekly computer lab practical sessions provided training fundamentals of R programming language, the R-studio integrated development environment (IDE), and the GitHub.com open-source software development platform. RESULTS/ANTICIPATED RESULTS: The graded assignment consisted of a coding project in which students were each assigned an individual parameter from the CBC results. These include, for example, relative lymphocyte count or hemoglobin readouts. Students each created their own R-language script using R-studio, with functional code which: 1) Read in data from a file provided, 2) Performed statistical testing, 3) Read out statistical results as text, and charts as image files, 4) “Diagnosed” individuals in the dataset as being inside or outside the clinical normal range for that parameter. Each student also registered their own GitHub account and published their open-source code. Grading was performed on code functionality by downloading each student repository and running the code with the instructor as an outside developer using the resource. DISCUSSION/SIGNIFICANCE OF IMPACT: In this curriculum, students with no background in programming learned to code a basic R-language script and use GitHub to automate interpretation of CBC results. With advanced automation now becoming commonplace in translational science, such course content can provide introductory level of literacy in development of clinical informatics software.

